# Factors affecting medical students’ attendance on clinical attachments: a qualitative focus group study

**DOI:** 10.1186/s12909-026-08672-3

**Published:** 2026-02-06

**Authors:** Richard T. Dalton, Dominic W. Proctor, Meghna Rao, Kunika Kakuta, William Coppola, Stella Ivaz, John Hines, Paul Dilworth

**Affiliations:** 1 Medical School Office, Royal Free Hospital, Royal Free London NHS Foundation Trust, Pond Street, London, NW3 2QG UK; 2 University College London Medical School, London, UK

**Keywords:** Attendance, Clinical attachment, Qualitative, Medical student

## Abstract

**Background:**

Recent literature has reported declining attendance among certain medical student cohorts. The limited existing studies primarily explore attendance at lectures but not attendance on clinical attachments, which are mandatory for satisfactory student progression. This study aimed to investigate the factors influencing medical students’ decision to attend learning activities on clinical attachments, to optimise attendance and to formulate recommedations with respect to educational outcomes and the student experience.

**Methods:**

Nine focus groups with clinical year medical students were conducted at a UK medical school, based at three different hospital sites (*n* = 39). Thematic analysis of transcripts was performed (NVivo Software V14.23.2).

**Results:**

Key factors which influenced medical students’ decision to attend clinical attachments were grouped into 6 main themes: (1) student factors, (2) learning activity factors, (3) assessment pressure, (4) organisational factors, (5) tutor factors, and (6) attendance monitoring. Fourteen sub-themes were then identified under these key themes. Specific factors that encouraged attendance included clustered learning activities that maximised student time efficiency and high tutor interactivity with an awareness of student-specific learning outcomes. Factors that discouraged attendance included a prioritisation of students for self-directed learning largely driven by exam pressure and a belief amongst some students that attendance on clinical attachments may not correlate with exam performance. Attendance may be optimised through clustering timetabled learning activities, transparently balancing self-study and timetabled activities, increased faculty development initiatives and further optimising university assessments to emphasise the skills acquired through patient exposure.

**Conclusions:**

This study highlights the heterogenous decision-making relating to attendance among medical students on clinical attachments. The data presented may help to explain the declining attendance trends on clinical attachments observed in certain cohorts. Future research could quantitatively investigate the effect of specific recommendations to optimise both attendance and the student experience.

## Introduction

Attendance across higher education institutions (HEIs) has long been debated in the educational literature [1]. Educational, moral, financial and reputational priorities may incentivise attendance irrespective of course subject; including the positive correlation between educational attainment and attendance identified in some studies, the correlation between reduced student interactivity and higher drop-out contemplation rates, and the detrimental ramifications for an organisation’s reputation if students graduate despite high rates of absenteeism [2–6]. However, the causal nature of the relationship between attendance and observable educational outcomes in all academic disciplines remains unclear. Several studies have investigated the factors influencing student attendance in HEIs more widely, which include a combination of individual (e.g. illness, family emergencies) and institutional factors (e.g. scheduling challenges, teaching issues, policy considerations) [1]. However, qualitative data investigating these factors among clinical medical student cohorts is scarce, resulting in challenges for stakeholders responsible for designing attendance policy.

Recent longitudinal data has demonstrated declining attendance among certain international medical school cohorts [7]. There are many possible explanations for this trend ranging from distance and blended learning techniques [8, 9], to rising levels of burnout among medical students [10]. Previous attempts at mandating attendance amongst medical students in an attempt to optimise educational outcomes have instead had the opposite effect; leading to students feeling infantalised and a breakdown of trust between the faculty and student body [11]. Thus the decision-making relating to the attendance of medical students is complex and currently poorly understood, However, the factors underpinning this is of particular importance in clinical attachments where reduced exposure to the hidden curriculum may result in professional development challenges for students [12]. Reduced exposure to the clinical environment may also hinder clinical competence, which has potentially significant implications for patient safety [13].

Given that the curriculum structure and associated stressors in medical curricula differ from most university courses, it is possible that different factors drive attendance decision-making among medical students. At present, existing studies have mainly investigated factors influencing medical student attendance at lectures rather than clinical attachments [14–17]. Therefore, this study aimed to explore the factors influencing medical students’ attendance on clinical attachments with a view to explaining the recently observed attendance trends and developing recommendations to optimise attendance policy.

## Methods

### Study design

This study was informed by a constructivist research paradigm. A focus group study was designed to address a lack of qualitative data in the current medical literature. Focus groups were preferred to interviews to explore the interpersonal dynamics underpinning attendance-related decision-making, and to improve the breadth of reported contextual insights [22]. Participants were recruited through representative sampling from students in their first and second clinical years (fourth and fifth years of undergraduate study) at three different hospital sites via email correspondence. Pre-clinical students were excluded having not undertaken clinical attachments, and final (sixth) year students excluded due to substantial differences between the structure of learning activities on clinical attachments (e.g. a greater focus on clinical responsibility).

### Data collection

Overall, nine face-to-face focus groups with clinical year medical students were conducted at a large UK medical school (*n* = 39; 27 female, 12 male; 27 fourth year, 12 fifth year). Median focus group attendance was four students (IQR: 4–5), with each focus group conducted by two members of the research team (a facilitator and moderator). Focus groups concluded at the point of theoretical sufficiency as determined by agreement of all members of the research team, meaning that no substantial contextual additions were made during focus groups. Questioning sought to elicit both factors that encouraged and discouraged attendance at learning activities on clinical attachment, pre-defined as small-group tutorials, bedside teaching, ward rounds, clinics, multi-disciplinary team meetings and other ad hoc activities occurring during clinical attachment timetabled weeks. Large-group lecture attendance was not explored with qualitative data already widely reported. The full question framework based on a collaborative exploratory literature search performed by the research team may be found in Appendix 1. Much of the existing literature relates to individual factors in non-medical programmes, hence during this review university factors were used as the basis of discourse to optimise relevance to stakeholders. A range of factors were investigated simultaneously to avoid over-inflation of any single factor [23]. A pilot focus group was conducted with students from the cohorts in question. Focus groups were recorded using a secure video-conferencing platform on an encrypted recording device and lasted between 45 and 60 minutes.

### Data analysis

Recordings were transcribed verbatim and thematic analysis was performed in line with Braun and Clarke’s method, using NVivo Software V14.23.2 (QSR International, Burlington MA) [24]. Each transcript was initially independently coded by two members of the research team as a means of sense checking. Each code comprised a sentence or phrase to denote the simplest elemental unit of meaning. These codes were later refined into themes at regular research team meetings between three members of the research team (RD, DWP, KK). Finally, relevant themes were divided into sub-themes based on the most substantive data. These sub-themes reflected patterns in students’ self-reported views, intentions and *anticipated* behaviours, as it was beyond the scope of this qualitative analysis to measure empirical attendance data.

### Ethics

Ethical approval was granted by the ethics committee at University College London (ID: 26277/001). This study is reported in line with Consolidated Criteria for Reporting Qualitative Research (COREQ) guidance [25] and conducted in accordance with the Declaration of Helsinki. Recordings and transcripts were stored on secure institutional servers in accordance with data protection protocols.

### Reflexivity

RTD, DWP and MR were clinical teaching fellows at the time of the study. PD, WC, JH and SI are senior medical school faculty and KK is a medical school Trust Liaison Officer. Having completed or been heavily involved with the organisation of undergraduate medical training, a variety of perspectives on the research topic existed with regular team meetings serving to mitigate researcher bias that may have arisen from pre-existing individual perceptions.

### Study context

In the United Kingdom (UK), undergraduate medical programmes typically span 5–6 years. While program structure varies slightly between institutions, medical students generally complete two years as ‘pre-clinical’ students, whereby there is a greater focus on anatomy, physiology and pathology delivered via lectures and small group learning, with limited clinical exposure. Following this, student learning is based predominantly in healthcare environments with supervised participation in patient care across different specialties – termed clinical attachments. While there is no exact directive about the nature of these attachments, satisfactory attendance and performance in workplace based assessments is mandatory for progression.

This study was conducted between December 2023 – January 2024 in the years following the COVID-19 pandemic. This time has seen a substantial increase in the implementation of distance learning techniques across healthcare disciplines [18–20]. Secondly, recent years have seen a greater reliance on self-directed study and multiple choice question banks as a key component of preparation for medical school assessments [21]. Thirdly, this study was conducted during a period of industrial action by UK resident doctors.

## Results

**Fig. 1:**
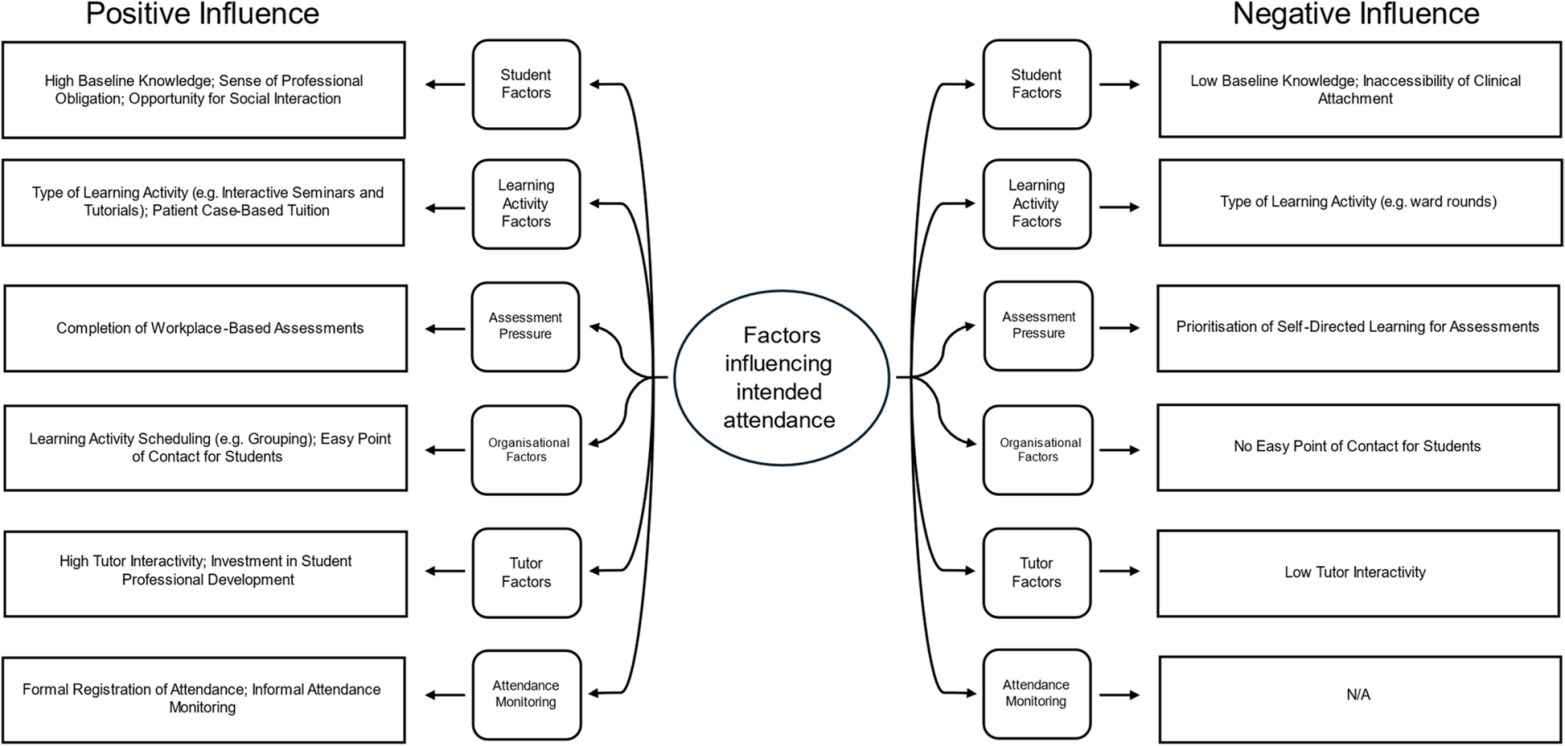
Summary of factors positively and negatively influencing attendance on clinical attachments

During research team meetings, thematic codes were refined into 6 main themes: (1) student factors, (2) learning activity factors, (3) assessment pressure, (4) organisational factors, (5) tutor factors, and (6) attendance monitoring. Fourteen subthemes were identified (Fig. 1) and there were no substantial differences in the factors reported by the two participating cohorts.

### Student factors

#### Baseline knowledge

Students reported that possessing greater baseline knowledge prior to a learning activity, facilitated a greater grasp of new learning content and resulted in a higher likelihood of attending. Conversely possessing a lack of knowledge, was a barrier to the students’ engagement and resulted in reduced likelihood of attendance.

- Participant 29: “*I find that if I don’t know content*,* it’s not as valuable coming in for stuff*,* so I like to like to learn it beforehand and then consolidate it when I come into clinical attachments*”.

#### Sense of professional obligation

Some students reported a sense of professional obligation to attend clinical attachments, with reference to the high level of responsibility encountered as a resident doctor. Links were also made by the students to the non-theoretical knowledge that may be gained from clinical attachments (such as communication, empathy and team working skills). This sense of professional obligation resulted in an increased likelihood of attendance.

- Participant 19: *“Half of my firm don’t come in*,* but when they do*,* I see that I might not know as much as them in terms of - I do worse on [question banks]*,* but I can speak to patients better and I can speak to doctors better. So I do value it*,* which is one of the reasons I turn up because medicine is not a memorization subject. It is more than that*,* it is vocational.”*

#### Opportunity for social interaction

Several students reported that the opportunity for social interaction with peers was a factor that increased their likelihood of attending clinical attachment. Students reported a sense of wellbeing when attending clinical attachments with friends and a sense of increased accountability if their friends were also expecting them to attend.

- Participant 10: “*I would say that people in your firm is quite important. Like if you get paired with people who are quite proactive… then you feel a pressure to kind of come in as well and not be lazy.”*

#### Inaccessibility of clinical attachment

Students that were required to travel long distances were less likely to attend, especially if the time and expense of travelling outweighed their personal preconceived educational benefit from the learning activity.

### Learning activity factors

#### Type of learning activity

Students were more likely to attend small group tutorials and clinical skills sessions compared to more ‘passive’ learning activities such as attending ward rounds and multidisciplinary team meetings. Reasons for this included higher levels of tutor interactivity and focused learning outcomes with smaller group tutorials.

Some students also noted they were less likely to attend tutorials both in-person and online as they felt pressured when asked to interact with the tutor in the presence of a large group of their peers. Tutor interactivity was generally positively perceived but only when experienced in a smaller group setting.

#### Patient case-based tuition

Students were more likely to attend clinical attachments if there was an opportunity for interaction with real patients to clinically contextualise theoretical knowledge. Examples given included clerking a new hospital admission and presenting to a tutor and attending bedside teaching.

### Assessment pressure

#### Self-directed learning

Students reported the need to balance attendance at clinical attachments to develop new clinical skills, with a need to undertake self-directed learning to ensure success in applied knowledge assessments. Specifically regarding written examination, several students perceived a negative correlation between examination scores and attendance on clinical attachments. This was frequently perceived as being a factor that reduced the likelihood of attendance.

- Participant 23: *“So everything’s been online for us since first year and we did OSCEs [Objective Structured Clinical Examinations] in second year after doing the whole year online anyway*,* and we all passed and did well. And so we were kind of like*,* OK*,* so then why do we need to be in every single day? Like what are we actually gaining that will help us for the OSCEs?”*
- Participant 30: *“In [hospital name] last year*,* I would come in for a ward round in the morning. The doctors wouldn’t talk to me. I would have to just walk behind them as they’re doing their ward rounds or again sitting in the corner during clinics. And it was a very demotivating because I felt like I could spend my time more wisely*,* for example*,* studying the material in the library and as opposed to just sitting and observing.”*

#### Workplace-Based assessments (WBAs)

Towards the end of the modules and the end of the academic year, it was reported that a need to complete WBAs and portfolio sign-offs positively influenced student intentions to attend learning activities on clinical attachment, with a view to meeting requirements for progression to the next year of study.

- Participant 10: “*If I come into hospital*,* I want to see a patient*,* I want to get DOPs [Direct Observation of Clinical Skills] or Mini-CEX [Mini-Clinical Evaluation Exercise] or something done”*.
- Participant 16: *(Opportunity for sign offs) does have an impact on attending… specifically to get clinical activity forms signed. Turn up to full clinics and then not come up to more clinics just because you’ll do the bare minimum to get the sign. Yeah. Like*,* come in*,* take a history and then leave.*

### Organisational factors

#### Learning activity scheduling

Students frequently reported that well-organised attachments, where timetables were issued in advance and an induction occurred, resulted in an increased likelihood of attendance.

- Participant 16: *“[Doctors] have to try and fit us in for an hour*,* which*,* if they just get a load of jobs to do*,* they can’t do. I might not go to a bedside teaching if I’d say been on the ward all morning because it’s going to start at least half an hour late because understandably*,* they’re busy. It’s not their only job.”*

Conversely, last minute learning activity cancellations, or learning activities occurring in multiple geographical locations within the same day reduced the likelihood of attendance.

- Participant 7: *“sometimes you’ve already planned things in advance*,* and then they’ll randomly add a teaching session and I can’t cancel what I’ve already planned. So you prioritise that over your teaching.”*

#### Point of contact for students

The lack of a clearly identifiable point of contact for each attachment (e.g. for late alterations), reduced the likelihood of attendance, conversely a clear point of contact for students, with thorough communication, increased the likelihood of attendance.

- Participant 12: *“[If the attachment team] is engaged with you… like they send emails that seem organised then it seems like it’s going to be a valuable way to spend your time.”*
- Participant 23: *“I think the reason why a lot of people don’t show up is because the doctor’s name on the timetable is not correct. We had emailed them and it came up with that automatic – ‘I’m on annual leave till this day’ reply and so we didn’t know the teaching was happening. We’d emailed them we just hadn’t heard anything back from anyone. So then people were like*,* OK*,* I’m not going to come in then.”*

### Tutor factors

#### Tutor interactivity

Students reported that high levels of tutor interaction increased their motivation to attend other learning activities on clinical attachment. Conversely, a lack of tutor interaction reportedly reduced students’ intentions to attend. Some students commented that low staffing levels negatively affected their motivation to attend clinical attachment. These sentiments were shared by most study participants.

- Participant 15: *“I think we take (attending attachments in the operating theatre) with a pinch of salt… most of the time*,* we know we’re not going to learn anything in there and no one’s going to speak to us.”*
- Participant 30: *“if they have a teaching fellow that is very active in communicating with the students*,* then I know that going to that clinic is going to provide me with more teaching*,* because the doctor will interact with the student more and teach the student as we go along.”*

#### Investment in student professional development

Students reported they felt more likely to attend sessions with tutors who had demonstrated an interest in student progression and in the curriculum’s intended learning outcomes. This related to explicitly setting clear learning outcomes and ensuring that content was appropriate to the student’s level of knowledge, which led to a sense of personal value.

- Participant 1: *“The teaching was just so good. I think I attended everything. Because everything was so interesting*,* and they were really invested in teaching us”*.
- Participant 8: *“It varies that there’s some people who are obviously passionate about teaching and all those who just turned up and like*,* say whatever is on their mind. If it’s going to be someone who isn’t that passionate about teaching then you would be less inclined to worry about letting them down. But if it is someone who really put lots of effort into teaching*,* then you want to go.”*

### Attendance monitoring

#### Formal registration of attendance

Students were more likely to attend if there was perception that a learning activity was more likely to be formally registered. Typically, this included small group tutorials and clinical skills sessions. The main reason for this was the potential for disciplinary repercussions. In comparison, students did not expect learning activities such as ward rounds to be registered which generally reduced their likelihood of attending these activities.

- Participant 24: *“Definitely when you have to sign your name or tick your name off*,* that is the reason why people come in*,* not necessarily just for the learning*,* it’s just for the percentage of their attendance.”*
- Participant 28: *“I think (attendance monitoring) does motivate you to go more*,* because you’re obviously scared of the consequences*,* but in some cases I don’t think it’s really warranted to be that particular on [attendance].”*

#### Informal attendance monitoring

Students were more likely to attend if they perceived a specific attachment to designate a higher informal emphasis on attendance in order to pass the module.

- Participant 5: *“We were turned away from things quite a lot*,* just being told*,* we’re too busy*,* go away*,* kind of thing. And that meant that you just are less likely to attend future things because there’s no point.* W*hereas… [other specialties] were very focused on attendance*,* which meant that we were more likely to go.”*

## Discussion

This focus group study highlights the complex and heterogenous nature of clinical medical student decision-making regarding attendance at learning activities on clinical attachments. Several key themes emerged in relation to individual (student factors) and institutional factors (learning activity factors, assessment pressure, organisational factors, tutor factors, attendance monitoring), partly in keeping with the wider educational literature but with some key differences specific to a clinical medical curriculum. Such heterogeneity implies that decisions are not solely determined by academic requirements, but shaped by a broader set of educational and personal contexts.

There is overlap between the factors affecting attendance on clinical attachments and factors affecting attendance in the wider educational literature. Key considerations in this study were the scheduling of learning activities (repetitive timetables, inaccessible locations), perceptions of tutors, learning activity formats and a desire to satisfy attendance monitoring policy. These have all been reported as factors impacting students’ intention to attend timetabled learning activities (most frequently in lecture-based settings) [1]. Specifically, too few tuition hours or extended intervals between classes has been found to reduce attendance, helpful and engaged tutors have been shown to increase lecture attendance and mandating attendance is widely associated with improved attendance. However, this study also identified a number of key factors with context-specific implications for clinical attachments.

Firstly, students frequently reported a desire to preferentially undertake self-directed learning, rather than spend time in the clinical environment. This was driven by a combination of single best answer assessment pressure and a feeling that learning on clinical attachments would be unlikely to increase attainment in these assessments to the same extent. Several possible factors may explain this phenomenon. Distance and blended learning techniques have been shown to have a comparable impact on academic attainment when compared with traditional face-to-face teaching [1]. This approach may be more in-line with student learning preferences, particularly in cohorts that have undertaken a substantial amount of distance learning in pre-clinical years of the medical course [26]. Recent systemic changes in academia and healthcare may also have contributed. The introduction of university tuition fees may encourage the commodification of students’ time (by policymakers and students) resulting in a preference for learning opportunities directly relevant to quantifiable assessments as opposed to more holistic clinical competence [27]. Skill acquisition from the hidden curriculum (communication, understanding of clinical norms) may be neglected with this outlook, highlighting a need to incorporate regular assessment of holistic clinical skills to emphasise the importance of developing these proficiencies. A greater emphasis on both WBAs and clinical supervisors’ judgements of student performance with feedback from patients may encourage the development of these skills.

Secondly, students reported that long travel duration had a negative impact on their intentions to attend activities on clinical attachments, also citing the cost of travel as an influential factor. This is of particular interest to medical school stakeholders given the travel requirements in medical curricula may be greater than in other courses. While there is limited evidence suggesting that travelling > 30 min may actually improve attendance in some student cohorts, transport issues and location inaccessibility have been shown to reduce attendance at other learning activities in keeping with our findings [28, 29]. Existing literature suggests attendance can be dependent on the average number of lectures per day and the number of scheduled breaks [30, 31]. This is replicated in studies related to absenteeism amongst clinical and non-clinical degrees, where students are less likely to attend sessions after long breaks and when lectures are held in isolation [32, 33]. Cost considerations may be alleviated by prompt travel re-imbursement and the avoidance of overly busy or sparse days [30]. Additionally, General Medical Council guidance on undergraduate clinical attachments states that students should be empowered to pursue learning through other modalities including self-study, which offer an alternative means of reducing travel burden. These considerations highlight the importance of a student-stakeholder dialogue in determining the optimal proportion of different learning formats on clinical attachments.

Thirdly, it was frequently reported that students were less likely to attend activities delivered by tutors whose previous activities had lacked structure and interactivity. While the role of engaged tutors has previously been implicated in positively impacting attendance in lectures [34, 35], it has not been widely reported among medical students on clinical attachments. This presents a unique challenge for medical curriculum stakeholders. Unlike many other courses, clinical attachments often rely heavily on tutors that are not directly employed by the HEI overseeing curriculum delivery. As a result, it is possible that tutors may occasionally lack detailed knowledge of intended learning outcomes, which may partially explain our findings. Faculty development is a crucial component of medical curricula but is rarely employed among junior clinicians despite its apparent benefits [36, 37]. To address this issue, stakeholders may consider increasing the delivery of faculty development initiatives among clinical staff irrespective of seniority [38], or employing a greater number of doctors with contracted teaching time (commonly termed clinical teaching fellows) [39, 40].

Fourthly, the cancellation of sessions with little prior notice and poor attachment organisation were cited as factors that negatively impacted attendance. Students reported this was due to a lack of confidence that subsequent sessions would occur as scheduled. This negative effect has been demonstrated in a previous study, which identified reduced morale and a sense of instability amongst students following lecture cancellations [41]. These outcomes were compounded in scenarios in which students had incurred significant travel time to attend cancelled sessions. This highlights the necessity of accessible and reliable timetabling with a robust system to disseminate messages relating to the cancellation of sessions. Although some cancellations will be unavoidable, keeping students fully informed when cancellations occur encourages trust and maintains respect between HEIs and the student body, potentially encouraging future attendance.

### Strengths and limitations

The strengths of this study primarily relate to the comprehensive nature of the data collected that addresses a previously under-investigated aspect of medical education. The limitations, common to all focus group studies, include a susceptibility to researcher bias (somewhat mitigated by focus group moderation). While representative sampling was used to select study participants, ultimately participation was optional hence a degree of population bias may remain. Furthermore, participants were from a single medical school (but multiple hospital sites), potentially limiting the generalisability of the results.

## Conclusions

This study highlights the heterogenous nature of decision-making relating to attendance among medical students on clinical attachment. While there is overlap with existing attendance literature, several factors specific to medical student decision-making are described, most notably a preference to undertake self-directed learning rather than attending learning activities on clinical attachments and challenges surrounding a lack of tutor engagement. This study extends current knowledge by providing qualitative data that may explain the declining attendance trends on clinical attachments observed in certain medical cohorts. Furthermore, we have suggested some practical recommendations that medical schools may consider implementing to optimise their clinical curricula. Future research could explore the impact of the suggested recommendations on attendance and student satisfaction on clinical attachments.

## Data Availability

The datasets generated and/or analysed during the current study are not publicly available as this would compromise participant privacy. These are available from the corresponding author on reasonable request.

## Appendix

### Focus group questioning framework

Introduction: What factors influence your decision to attend clinical placement?

*Question 1: How important is the subject matter in influencing your decision to attend?*

Prompts: Does this relate to the title of the activity, your current level of knowledge, passing assessments, your previous experience of similar activities (or others’ experience), a desire to treat patients more effectively, or other factors?

Do you think there is a link between attendance at clinical placement and performance in assessment?

*Question 2: Does the style of learning activity affect your decision?*

Prompts: Does this relate to the duration of the activity, the time at which it is scheduled (e.g. twilight shift/day placement), the tutor running the session, the type of activity (workshop, placement etc.), or other factors?

*Question 3: How does the organisation of learning activities influence your decision?*

Prompts: For example, does this relate to the likelihood the session will be cancelled, the likelihood tutors will not arrive, the likelihood that other students will not attend, or other factors?

*Question 4: Does the opportunity for social interaction influence your decision?*

Prompts: For example, does this relate to interaction with peers, interaction with tutors, near-peer learning, or other factors?

*Question 5: Does the need to fulfil professional obligations influence your decision to attend?*

Prompts: For example, does this relate to a desire to not let people down (patients/tutors/peers), attendance monitoring, potential disciplinary action, General Medical Council attendance requirements, an internal sense of responsibility, or other factors?

## Abbreviations

COREQ: Consolidated Criteria for Reporting Qualitative Research
HEI: Higher Education Institution
OSCE: Objective Structured Clinical Examination
WBA: Workplace-Based Assessment

## References

[1] Moores E, Birdi GK, Higson HE. Determinants of university students’ attendance. Educ Res. 2019;61(4):371–87. Available from: https://research.aston.ac.uk/en/publications/determinants-of-university-students-attendance.

[2] Credé M, Roch SG, Kieszczynka UM. Class attendance in college: A meta-analytic review of the relationship of class attendance with grades and student characteristics. Rev Educ Res. 2010;80(2):272–95. Available from: https://journals.sagepub.com/doi/full/10.3102/0034654310362998.

[3] Webb OJ, Cotton DRE. Early withdrawal from higher education: a focus on academic experiences. Teach High Educ. 2018;23(7):835–52.

[4] St. Clair KL. A case against compulsory class attendance policies in higher education. Innov High Educ. 1999;23(3):171–80.

[5] Subramaniam B, Komattil R, Hande S. Attendance and achievement in medicine: investigating the impact of attendance policies on academic performance of medical students. Ann Med Health Sci Res. 2013;3(2):202. 23919190 10.4103/2141-9248.113662 PMC3728863

[6] Al Shenawi H, Yaghan R, Almarabheh A, Al Shenawi N. The relationship between attendance and academic performance of undergraduate medical students during surgical clerkship. BMC Med Educ. 2021;21(396). 10.1186/s12909-021-02833-2 PMC8298040 34294063

[7] AAMC. Year Two Questionnaire (Y2Q) 2020 All Schools Summary Report. 2021. Available from: https://www.aamc.org/data-reports/students-residents/report/year-two-questionnaire-y2q.

[8] Vallee A, Blacher J, Cariou A, Sorbets E. Blended learning compared to traditional learning in medical education: systematic review and meta-analysis. J Med Internet Res. 2020;22(8):e16504. Available from: https://www.jmir.org/2020/8/e16504. 32773378 10.2196/16504 PMC7445617

[9] Dhar P, Rocks T, Samarasinghe RM, Stephenson G, Smith C. Augmented reality in medical education: students’ experiences and learning outcomes. Med Educ Online. 2021;26(1):1953953. Available from: https://www.tandfonline.com/doi/epdf/10.1080/10872981.2021.1953953. 10.1080/10872981.2021.1953953 PMC8281102 34259122

[10] Ishak W, Nikravesh R, Lederer S, Perry R, Ogunyemi D, Bernstein C. Burnout in medical students: a systematic review. Clin Teach. 2013;10(4):242–5. Available from: https://onlinelibrary.wiley.com/doi/full/10.1111/tct.12014. 23834570 10.1111/tct.12014

[11] Lamb S, Chow C, Lindsley J, Stevenson A, Roussel D, Shaffer K, et al. Learning from failure: how eliminating required attendance sparked the beginning of a medical school transformation. Perspect Med Educ. 2020;9(5):314–7. Available from: https://link.springer.com/article/10.1007/s40037-020-00615-y. [cited 2024 Sep 13]. 32804346 10.1007/s40037-020-00615-y PMC7550439

[12] Neve H, Collett T. Empowering students with the hidden curriculum. Clin Teach. 2018;15(6):494–9. Available from: https://onlinelibrary.wiley.com/doi/full/10.1111/tct.12736. 29178606 10.1111/tct.12736

[13] De Lasson L, Just E, Stegeager N, Malling B. Professional identity formation in the transition from medical school to working life: A qualitative study of group-coaching courses for junior Doctors. BMC Med Educ. 2016;16(1):1–7. Available from: https://bmcmededuc.biomedcentral.com/articles/10.1186/s12909-016-0684-3. 27342973 10.1186/s12909-016-0684-3 PMC4919855

[14] Hoyo LM, Yang CY, Larson AR. Relationship of medical student lecture attendance with Course, Clerkship, and licensing exam scores. Med Sci Educ. 2020;30(3):1123. 34457774 10.1007/s40670-020-01022-y PMC8368765

[15] Mitra S, Sarkar P, Bhattacharyya S, Basu R. Absenteeism among undergraduate medical students and its impact on academic performance: A record-based study. J Educ Health Promot. 2022;11. Available from: https://pmc.ncbi.nlm.nih.gov/articles/PMC9942141. 10.4103/jehp.jehp_638_21 PMC9942141 36824099

[16] Sharmin T, Azim E, Choudhury S, Kamrun S. Reasons of absenteeism among undergraduate medical students: A review. Anwer Khan Mod Med Coll Jounral. 2016;8(1):60–6.

[17] Gardner G, Feldman M, Santen SA, Mui P, Biskobing D. Determinants and outcomes of In-person lecture attendance in medical school. Med Sci Educ. 2022;32(4):883. 35821745 10.1007/s40670-022-01581-2 PMC9264290

[18] Stoehr F, Müller L, Brady A, Trilla A, Mähringer-Kunz A, Hahn F et al. How COVID-19 kick-started online learning in medical education - The DigiMed study. PLoS ONE. 2021;16(9):e0257394. Available from: https://journals.plos.org/plosone/article?id=10.1371/journal.pone.0257394. 10.1371/journal.pone.0257394 PMC8454930 34547031

[19] Muhaimin M, Habibi A, Riady Y, Alqahtani TM, Chaerunisaa AY, Wijaya TT, et al. Covid-19 distance and online learning: a systematic literature review in pharmacy education. BMC Med Educ. 2023;23(1):1–10. Available from: https://bmcmededuc.biomedcentral.com/articles/10.1186/s12909-023-04346-6. 37221539 10.1186/s12909-023-04346-6 PMC10204690

[20] Abdull Mutalib AA, Md. Akim A, Jaafar MH. A systematic review of health sciences students’ online learning during the COVID-19 pandemic. BMC Med Educ. 2022;22(1):1–34. Available from: https://bmcmededuc.biomedcentral.com/articles/10.1186/s12909-022-03579-1. 35786374 10.1186/s12909-022-03579-1 PMC9251028

[21] Wu JH, Gruppuso PA, Adashi EY. The Self-directed medical student curriculum. JAMA. 2021;326(20):2005–6. Available from: https://jamanetwork.com/journals/jama/fullarticle/2785917. 34724030 10.1001/jama.2021.16312

[22] Stalmeijer RE, McNaughton N, Van Mook KA, Stalmeijer WNE, Mcnaughton RE. Using focus groups in medical education research: AMEE guide 91. Med Teach. 2014;36(11):923–39. Available from: https://www.tandfonline.com/doi/abs/10.3109/0142159X.2014.917165. 25072306 10.3109/0142159X.2014.917165

[23] Dolnicar S, Kaiser S, Matus K, Vialle W. Can Australian universities take measures to increase the lecture attendance of marketing students? J Mark Educ. 2009;31(3):203–11. [cited 2024 Oct 3]. Available from: http://jmd.sagepub.comhttp//online.sagepub.com.

[24] Braun V, Clarke V. Using thematic analysis in psychology. Qual Res Psychol. 2006;3(2):77–101. Available from: https://www.tandfonline.com/action/journalInformation?journalCode=uqrp20.

[25] Tong A, Sainsbury P, Craig J. Consolidated criteria for reporting qualitative research (COREQ): a 32-item checklist for interviews and focus groups. Int J Qual Heal Care. 2007;19(6):349–57. 10.1093/intqhc/mzm042. 10.1093/intqhc/mzm042 17872937

[26] Ahmady S, Kallestrup P, Sadoughi M, Katibeh M, Kalantarion M, Amini M et al. Distance learning strategies in medical education during COVID-19: A systematic review. J Educ Health Promot. 2021;10(421). Available from: https://pmc.ncbi.nlm.nih.gov/articles/PMC8719547/. 10.4103/jehp.jehp_318_21 PMC8719547 35071627

[27] Naidoo R, Whitty G. Students as consumers: commodifying or democratising learning? Int J Chin Educ. 2014;2(2):212–40. Available from: https://journals.sagepub.com/doi/10.1163/22125868-12340022.

[28] Kottasz R. Reasons for student Non-Attendance at lectures and tutorials: an analysis. Investig Univ Teach Learn. 2005;2(2):5–16. Available from: chrome-extension://efaidnbmnnnibpcajpcglclefindmkaj/https://core.ac.uk/download/pdf/36771681.pdf.

[29] Kirby A, McElroy B. The effect of attendance on grade for first year economics students in university college Cork. Econ Soc Rev (Irel). 2003;34(3):311–26. Available from: http://www.tara.tcd.ie/handle/2262/61584.

[30] Larabi-Marie-Sainte S, Jan R, Al-Matouq A, Alabduhadi S. The impact of timetable on student’s absences and performance. PLoS ONE. 2021;16(6):e0253256. Available from: https://journals.plos.org/plosone/article?id=10.1371/journal.pone.0253256. 10.1371/journal.pone.0253256 PMC8232426 34170914

[31] Nevins EJ, Moori PL, Alexander L, Richards B, Bleasdale V, Sharma AK. Could attendance at medical school be improved? A prospective study of medical education at the university of liverpool: study of attendance at a UK medical school. MedEdPublish. 2016;5:78.

[32] Davis EA, Hodgson Y, Macaulay JO. Engagement of students with lectures in biochemistry and Pharmacology. Biochem Mol Biol Educ. 2012;40(5):300–9. Available from: https://pubmed.ncbi.nlm.nih.gov/22987551/. 22987551 10.1002/bmb.20627

[33] Kelly GE. Lecture attendance rates at university and related factors. J Furth High Educ. 2012;36(1):17–40. Available from: https://www.tandfonline.com/doi/abs/10.1080/0309877X.2011.596196.

[34] Bati AH, Mandiracioglu A, Orgun F, Govsa F. Why do students miss lectures? A study of lecture attendance amongst students of health science. Nurse Educ Today. 2013;33(6):596–601. 22863210 10.1016/j.nedt.2012.07.010

[35] Fjortoft N. Students’ motivations for class attendance. Am J Pharm Educ. 2005;69(1):107–12.

[36] Dijk SW, Findyartini A, Cantillon P, Cilliers F, Caramori U, O’Sullivan P, et al. Developing a programmatic approach to faculty development and scholarship using the ASPIRE criteria: AMEE guide 165. Med Teach. 2024. 10.1080/0142159X.2023.2259062. https://www.tandfonline.com/doi/abs/. 10.1080/0142159X.2023.2259062 37783204

[37] Burgess A, van Diggele C, Mellis C. Faculty development for junior health professionals. Clin Teach. 2019;16(3):189–96. Available from: https://onlinelibrary.wiley.com/doi/full/10.1111/tct.12795. 29790658 10.1111/tct.12795

[38] Hewson MG, Copeland HL, Fishleder AJ. What’s the use of faculty development? Program evaluation using retrospective Self-Assessments and independent performance ratings. Teach Learn Med. 2001;13(3):153–60. Available from: https://www.tandfonline.com/doi/abs/10.1207/S15328015TLM1303_4. 11475658 10.1207/S15328015TLM1303_4

[39] Harris IM, Greenfield S, Ward DJ, Sitch AJ, Parry J. The clinical teaching fellow role: views of the heads of academy in the West Midlands. BMC Med Educ. 2023;23(1):1–9. Available from: https://bmcmededuc.biomedcentral.com/articles/10.1186/s12909-023-04219-y. 37060013 10.1186/s12909-023-04219-y PMC10103451

[40] Qureshi S. Teaching fellowships for UK foundation Doctors. Med Teach. 2015;37(1):90–1. Available from: https://www.tandfonline.com/doi/abs/10.3109/0142159X.2014.916781. 25073019 10.3109/0142159X.2014.916781

[41] Drew S. Student perceptions of what helps them learn and develop in higher education. Teach High Educ. 2001;6(3):309–31.

